# Patient classification as an outlier detection problem: An application of the One-Class Support Vector Machine

**DOI:** 10.1016/j.neuroimage.2011.06.042

**Published:** 2011-10-01

**Authors:** Janaina Mourão-Miranda, David R. Hardoon, Tim Hahn, Andre F. Marquand, Steve C.R. Williams, John Shawe-Taylor, Michael Brammer

**Affiliations:** a Department of Neuroimaging, Centre for Neuroimaging Sciences, Institute of Psychiatry, King's College London, UK; b University of Würzburg, Würzburg, Germany; c Department of Computer Science, Centre for Computational Statistics and Machine Learning, University College London, UK; d Department of Cognitive Psychology II, Johann Wolfgang Goethe University Frankfurt/Main, Germany

**Keywords:** fMRI, Pattern classification, Depression, Machine learning, Support Vector Machine, Outlier detection

## Abstract

Pattern recognition approaches, such as the Support Vector Machine (SVM), have been successfully used to classify groups of individuals based on their patterns of brain activity or structure. However these approaches focus on finding group differences and are not applicable to situations where one is interested in accessing deviations from a specific class or population. In the present work we propose an application of the one-class SVM (OC-SVM) to investigate if patterns of fMRI response to sad facial expressions in depressed patients would be classified as outliers in relation to patterns of healthy control subjects. We defined features based on whole brain voxels and anatomical regions. In both cases we found a significant correlation between the OC-SVM predictions and the patients' Hamilton Rating Scale for Depression (HRSD), i.e. the more depressed the patients were the more of an outlier they were. In addition the OC-SVM split the patient groups into two subgroups whose membership was associated with future response to treatment. When applied to region-based features the OC-SVM classified 52% of patients as outliers. However among the patients classified as outliers 70% did not respond to treatment and among those classified as non-outliers 89% responded to treatment. In addition 89% of the healthy controls were classified as non-outliers.

## Highlights

► It is possible to define a “normality boundary” based on patterns of fMRI from healthy subjects. ► Most severely depressed patients should lie outside this boundary. ► The amount of departure from the “normality boundary” is related to the severity of the depression.

## Introduction

Defining normative data is extremely important in clinical and cognitive neuroscience. In these contexts, normative data represent the range of performance of a group of healthy individuals with relatively homogeneous characteristics on a particular test or cognitive task. These normative reference groups are considered the “gold standard” against which an individual's performance is compared and contrasted (Mitrushina et al., 1999). In a clinical context, the concept of normative data is quite fundamental as the concept of mental illness carries with it the implicit recognition of a “normality” of behavior from which an individual has a detectable “distance”. The definition of such a distance is controversial, difficult and confounded by debate over whether and when non-normality should be considered an illness. Though such definitions are common in the context of psychometrics they are not often used in the area of neuroimaging.

Most neuroimaging studies have focused on describing statistically significant differences in brain activation (fMRI) due to a cognitive task or group membership (e.g. task 1 vs. task 2 or patients vs. healthy controls) or in gray/white matter density (sMRI) due to a group membership (e.g. group 1 vs. group 2). These approaches can reveal where the alterations are located in the brain but in clinical applications this information cannot easily be used to aid an individual's diagnosis of single new subjects. Recently, pattern recognition classification techniques have sought patterns of brain activation that distinguish between cognitive states (e.g. Mourão-Miranda et al., 2005; Haynes and Rees, 2006; Norman et al., 2006) or between healthy individuals and patients with psychiatric or neurological disorders (e.g. Fu et al., 2008; Marquand et al., 2008). In these applications brain scans are treated as spatial patterns and statistical learning methods are used to identify statistical properties of the data that discriminate between groups of subjects. Once the discriminative pattern is found it can be used to classify new subjects. These approaches represent an important paradigm shift in neuroimaging data analysis towards a more direct application of neuroimaging in clinical practice.

However none of these studies addresses the problem of measuring departures from a distribution of “normal” or “typical” patterns of brain activation or anatomy. In the present paper, we present a framework that models the boundary of this distribution of “normal patterns” based on whole-brain volumes of neuroimaging data and allows quantitative assessment of departures from this distribution. From a technical point of view, the problem of defining a distribution of typical (or normal) patterns of brain activation is much more complex than defining normative data based on a single or multiple psychometric scales. When based on a univariate measure it might be conceptualized as the individual being a certain number of standard deviations from the “normal” mean distribution. Using multivariate measures, for example in psychometrics, analogous measures of departure from normality can be proposed, such as Euclidian or Mahalanobis distances from a multivariate mean in a multidimensional metric space. The extremely large number of dimensions characteristic of neuroimaging data (hundreds of thousands of voxels) makes the application of standard statistical approaches difficult or impossible due to the small sample size that characterizes neuroimaging datasets. Therefore, we suggest the use of a procedure known as one-class classification. In contrast with normal classification problems where one tries to distinguish between two or more classes, one-class classification seeks to describe properties of a specific class and distinguish it from “novel” or outlier examples. One way of addressing this problem is to compute the probability density of a specific class and when a new example falls below some density threshold this new example is considered abnormal. However computing the probability density in neuroimaging can become difficult due to the extremely high dimensionality of the data and the small sample sizes normally available. Alternatively, one can use methods that compute only a boundary decision and do not rely on density estimation, such as the One Class Support Vector Machine (OC-SVM, Schölkopf et al., 2001). The OC-SVM computes a decision boundary with the minimal volume around a subset of examples of the target class (training examples). Once the decision boundary is computed it can be used to classify new test examples as outliers (if they fall outside the boundary) or non-outliers (if they fall inside the boundary). In addition, the distance from the test examples to the boundary can be used to quantify the degree of abnormality and therefore can be correlated with other clinical or psychological measures for validation.

As a proof of concept we applied the OC-SVM to define the boundary of a distribution of “normal” or “typical” patterns of brain activation to sad facial expressions and tested the performance of this boundary in classifying patterns of healthy controls and depressed patients. If the distribution of “normal” patterns of brain activity or response to a specific stimulus is homogeneous enough to enable the definition of a robust boundary, deviations from this boundary represent an objective measure of the degree to which a psychiatric disorder affects brain functions.

In the present work we applied the OC-SVM approach to investigate three hypotheses: (i) the pattern of fMRI response to sad faces in healthy subjects is homogeneous enough to enable the definition of a “normality boundary”; (ii) this pattern is altered in depressed patients and (iii) the amount of departure from the “normality boundary” as measured by the OC-SVM is related to the severity of the depression.

## Methods

### Subjects

Nineteen participants (13 women and 6 men; age range, 29–58 years) meeting DSM-IV criteria for major depressive disorder according to the Structured Clinical Interview for DSM-IV Axis I Disorders (First et al., 1995) and a clinical interview with a psychiatrist were recruited through local newspaper advertisements. Inclusion criteria were an acute episode of major depressive disorder of the unipolar subtype (DSM-IV) and a score of at least 18 on the 17-item Hamilton Rating Scale for Depression (HRSD) (Hamilton, 1960). Exclusion criteria were a history of neurological trauma resulting in loss of consciousness, current neurological disorder, current comorbid Axis I disorder, including bipolar disorder and anxiety disorder or a history of substance abuse within 2 months of study participation. All patients were free of psychotropic medication for a minimum of 4 weeks at recruitment. Nineteen healthy comparison subjects (11 women and 8 men matched by age and IQ) with HRSD scores of less than 8 and no history of any psychiatric disorder, neurological disorder, or head injury resulting in a loss of consciousness were recruited by advertisement from the local community. All participants provided written informed consent. The project was approved by the Research Ethics Committee, Institute of Psychiatry, London, England.

Subjects were recruited for a prospective fMRI treatment study of depression using the antidepressant medication, fluoxetine. The longitudinal fMRI data of the sad facial affect task have already been presented (Fu et al., 2004). Pattern classification using a standard two class SVM has also been previously applied to the baseline (week 0) data acquired while patients were acutely depressed and medication-free (Fu et al., 2008). The present study focuses on applying the OC-SVM to the baseline data (Table 1) as an outlier detection approach. Patients were classified as responders to the antidepressant medication if their HRSD at week 8 was below 10 otherwise they were classifiers as non-responders.

### Implicit sad facial affect recognition task

Ten faces (5 male) from a standardized series of facial expressions of sadness (Ekman and Friesen, 1976) were morphed to represent low, medium, and high intensities of sadness. In an event-related fMRI paradigm, facial stimuli and baseline trials (crosshair fixation) were presented in random order. Each facial stimulus was presented twice at each intensity of sadness (60 faces in total), along with 12 baseline trials (crosshair visual fixation point), giving a total of 72 trials. Each face was presented for 3 s, and the inter-trial interval was randomly varied according to a Poisson distribution with a mean value of 5 s. The total duration of the experiment was 360 s.

For each facial trial, subjects were asked to indicate the gender of the face (male or female) by lateral movement of a joystick; no hand movement was required in response to a baseline trial. This strategy was used to ensure engagement with the task while eliciting incidental or implicit affective processing. Further details of the experimental design and procedures can be found elsewhere (Fu et al., 2004).

### fMRI data acquisition

Gradient-echo single-shot echoplanar imaging was used to acquire BOLD T2*-weighted image volumes on a neuro-optimized 1.5-T IGE LX System (General Electric, Milwaukee, Wis) at the Maudsley Hospital, South London, and Maudsley NHS Trust, London. We acquired 180 volumes for the sad facial affect task. For each volume, 16 noncontiguous axial slices parallel to the intercommissural plane were collected with the following parameters: repetition time, 2000 ms; echo time, 40 ms; section thickness, 7 mm; section skip, 0.7 mm; and in-plane resolution, 3 × 3 mm. To facilitate registration of the fMRI data in standard space, we also acquired a 43-slice, high-resolution inversion recovery echo planar image of the whole brain parallel to the intercommissural plane with the following parameters: repetition time, 16,000 ms; echo time, 73 ms; inversion time, 180 ms; and section thickness, 3 mm.

### Data preprocessing and representation

The fMRI data were realigned to remove residual motion effects, transformed into standard space (Talairach and Tournoux, 1988), and smoothed in space using an 8 mm Gaussian filter (FWHM), using SPM2 (Wellcome Department of Imaging Neuroscience, London, UK). For each subject, a General Linear Model (GLM) was implemented in SPM2 in which the effect of each condition was modeled by the convolution of the events with a standard hemodynamic response function (i.e. each condition corresponded to a regressor in the GLM model). We used two different approaches to define the features: voxel-based features and region-based features. In the first approach the images corresponding to the coefficients computed for each regressor were the spatial patterns of brain activation (whole brain voxel features). In the second approach we used a predefined anatomical template (Automated Anatomical Labeling, AAL template, Tzourio-Mazoyer et al., 2002) and we average the coefficients values within each regions to create a feature vector based on the regions (whole brain regional features). In both approaches we concatenated the task components.

### Pattern recognition analysis and kernel methods

The aim of pattern recognition analysis is to study general types of relations in the data that can be used to take actions such as classification, regression, clustering, etc. Recently a class of algorithms known as kernel methods has been developed for pattern analysis (e.g. Vapnik, 1995; Shawe-Taylor and Cristianini, 2004). Kernel methods are based on a pairwise similarity measure between all data sample or patterns, called a kernel matrix, which can be linear or non-linear, therefore these approaches can efficiently explore linear as well as non-linear relationships in the data. Using a nonlinear kernel matrix is equivalent to mapping the data from the original *input space* into a high dimensional *feature space* where the separation between the two classes can be easier (i.e. linear boundary). In addition kernel methods enable us to use a dual formulation for regression and classification models, i.e. we can express the problem in terms of the number of samples instead of number of dimensions. Using the dual formulation with proper regularization enables the solution of ill-conditioned problems (e.g. when the dimensionality is greater than the number of examples). The crucial observation about the dual formulation is that the information from the training examples is given by the inner products between pairs of training points. The regularization is used to restrict the choice of functions when there is not enough information in the data to precisely specify the solution. (For a more thorough introduction to kernel methods please see Chapter 2 of Shawe-Taylor and Cristianini, 2004).

### Classification problems: two classes vs. one class approach

In the context of pattern classification of brain images, each spatial pattern (e.g. whole-brain fMRI scan) corresponds to a point in the input space and each voxel in the brain image represents one dimension of this space. In a standard two-class problem, during the training phase the pattern recognition algorithm (such as the two class SVM) finds the hyperplane or decision function that separates the examples in the input or feature space according to the class label. The decision function thus represents a discriminative boundary between the two classes. Once the decision function is determined from the training data, it can be used to predict the class label of a new test example. In contrast with normal two-class classification problems, one-class classification seeks to describe properties of a specific class, and to distinguish it from examples considered outliers. During the training phase, the algorithm (e.g. the one-class SVM) computes a decision boundary that encloses most of the training examples. In this case the decision function is related to properties of a specific class (i.e. the positive class that was used for training) and not with discrimination between two classes. Once the decision boundary is computed it can be used to classify new test examples as outliers (if they fall outside the boundary) or non-outliers (if they fall inside the boundary). In addition the distance from the test examples to the boundary can be used as a measure of atypicality or abnormality. The OC-SVM solves therefore a more general (and difficult) problem than conventional two-class SVM.

In summary, two-class and one-class classification approaches address fundamentally different questions. The first finds the discriminative boundary between two classes and the second finds the boundary enclosing a specific class in relation to which patterns belonging to other classes can be detected as outliers. In practice, if one is interested in training a classifier to discriminate two well-defined and homogeneous classes with high accuracy, the standard two class SVM will have the best performance. However the one-class approach will be more advantageous in situations where one is interested in separating two (or more) classes, one of them being more homogeneous and well defined and the other(s) being highly heterogeneous and/or with small sample sizes.

### One Class Support Vector Machine (OC-SVM)

**Fig. 1:**
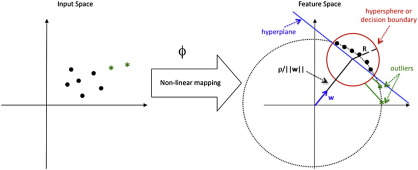
Illustration of the OC-SVM with RBF kernel. In this case finding the smallest hypersphere enclosing the data (red circle) is equivalent to finding the hyperplane that separates the data from the origin with maximal margin (blue line). The distance between the hyperplane and the origin is ρ/||w||, where w is the normal vector to the hyperplane and r is the offset. R corresponds to the radius of hypersphere.

The OC-SVM is a special case of the SVM algorithm for novelty or outlier detection (Schölkopf et al., 2001; Shawe-Taylor and Cristianini, 2004). The purpose of the OC-SVM algorithm is to estimate a decision function or boundary *f*(**x**) that takes the value + 1 in a small region capturing most of the training examples, and − 1 elsewhere. The OC-SVM algorithm involves mapping the data into a kernel or feature space and finding the smallest hypersphere that contains most of the training data. For kernels that depend only on the Euclidian distance between two patterns there is a correspondence between hyperspheres and hyperplanes in the kernel space, i.e. finding the smallest hyphersphere containing most of the data is equivalent to separating the data from the origin with the maximum margin (Fig. 1). The same equivalence holds if the data are normalized as they can be viewed as lying on the surface of a (unit) hypersphere in the feature space (Shawe-Taylor and Cristianini, 2004).

For a new example **x**, the value *f*(**x**) is determined by evaluating whether **x** it falls inside or outside the hypersphere. In the present study **x** represents a spatial pattern of brain activation. We used a Gaussian (or Radial Basis Function, RBF) kernel to map the data into the feature space (please refer to Appendix material for the RBF formulation). The RBF kernel is non-linear and therefore there is no unique mapping from the feature space back to the input space, this is known as the preimage problem in kernel methods (Schölkopf and Smola, 2002). In contrast with the linear kernel classifiers where one can plot directly the classifier's weight vector as a brain image showing the relative weight of each voxel for the decision function (Mourão-Miranda et al., 2005), in the non-linear case plotting the classifier's weight vector is not straightforward and its interpretation is often non-intuitive and complex. However some procedures have been proposed to generate maps for non-linear classifiers. Schölkopf et al. (1999) proposed an algorithm to generate preimages by approximating the inverse mapping from the feature space to the input space. Kjems et al. (2002) proposed an approach to derive a sensitivity measure based on the derivatives of the decision function with respect to its arguments. Lao et al. (2004) proposed an alternative approach to generate a spatial map based on the gradient of the decision function. For a given pattern of brain activation, following the gradient of the decision function gives the fastest path that will “make a healthy brain look like an abnormal brain”. In the present work we used the approach proposed by Schölkopf et al. (1999) to approximate the preimages for the OC-SVM with RBF kernel. The OC-SVM weight is a spatial representation of the decision boundary, which is based only on the class used for training (i.e. voxels with higher values have higher contributions to the classification). Here we present the OC-SVM weight maps to provide an intuition on the relative weight of the features (voxels or regions) to the decision function (i.e. to detect outliers) however those maps should not be interpreted as statistical tests describing activations. Please refer to Appendices A and B for a summary of the OC-SVM formulation and the pre-image approximation, respectively.

### Statistical map based on the positive class

In order to compare the OC-SVM maps with the statistical maps based on the positive class we applied one-sample t-test to the same data used as input to the OC-SVM (beta images for each subject and each condition). This analysis corresponds to a standard second level univariate statistic in fMRI. In the first stage, each subject's data were analyzed individually using a GLM. This produced images with the regression coefficients for each experimental condition (i.e. beta images). In the second stage, for each experimental condition, we applied one-sample t-test to test the null hypothesis that the mean coefficient value was equal to zero, i.e. there was no effect of the experimental condition (for details see Appendix C).

We used the same data to create the t-maps (statistical maps based on the positive class) and as input to the OC-SVM therefore the differences observed on the maps will not be due to pre-processing procedures. The OC-SVM is a multivariate approach and solves an optimization problem (i.e. estimate a decision function or boundary that takes the value + 1 in a small region capturing most of the training examples, and − 1 elsewhere) and the t-test is a univariate statistical approach applied to test the null hypothesis that the mean coefficient value for a specific voxel is zero (i.e. there is no effect of the experimental condition).

### Training and parameter optimization procedures

In order to test the hypothesis that the pattern of brain activation in response to sad emotional stimuli is abnormal in depressed patients, we trained an OC-SVM classifier using only the patterns of brain activation of healthy subjects in response to sad facial expressions (positive class) and tested whether the patterns of response of depressed patients (negative class) would be detected as outliers. Spatial patterns were defined by combining the patterns of brain response for the three intensities of emotional expression (low, medium and high intensity of sadness) into a single vector. We used two different approaches to define the features: voxel-based features and region-based features.

We used the ν-SVM implementation with Radial Basis Function (RBF) kernel. In order to optimize the kernel parameter (i.e. sigma in the RBF kernel) and the parameter ν that controls the outlier ratio we employed nested leave-one-out cross-validation procedure in two steps. We first excluded a healthy subject to comprise the test set, then we performed a second split where we repeatedly repartitioned the remaining 18 subjects into a validation set (1 subject) and training set (17 subjects). We repeated the internal leave-one-out split twice, first we kept the parameter ν fixed at an initial value (0.1) and chose the kernel parameter that optimized the accuracy on the internal leave-one-out and after we kept the kernel parameter fixed at the optimal value and chose the parameter ν that optimized the accuracy on the internal leave-one-out. We also tried to optimize the two parameters (ν and sigma) simultaneously, in this case the results were the same for the voxel-based analysis and slightly worse for the region-based analysis. If more than one value of the sigma resulted in the same accuracy we chose the smallest one. We did not optimize the threshold of the decision boundary (i.e. test examples with negative decision values are considered outliers). The OC-SVM was finally trained using the optimal parameters (ν and sigma) and tested with the healthy subject left out and a depressed patient, which was previously matched by age and gender to the healthy control subject. This procedure was repeated 19 times, each time leaving a different healthy subject outside as test subject (which was matched each time to a different patient).

We also repeated the same procedure using the patient group as the positive class in order to investigate if there was a consistent activation pattern among the patients in relation to which the controls would be classified as outliers.

In order to correlate the patients' test predictions with the HRSD and to generate the OC-SVM maps we re-trained the OC-SVM using data from all healthy control subjects (using the average parameters over the leave-one-out procedures) and tested all the patients using the trained classifier. This assures that the test predictions of different patients can be compared to each other as they were all computed in relation to the same classifier or decision boundary.

We used the LIBSVM toolbox (http://www.csie.ntu.edu.tw/~cjlin/libsvm/) and customized Matlab (http://www.mathworks.com/) codes to perform the analysis.

### Permutation test

We used a permutation test to compute a p-value for the True Positive ratio (percentage of patients detected as outliers by the classifier) and True Negative ratio (percentage of controls detected as non-outliers by the classifier) of the OC-SVM. Here, we permuted each group's labels 1000 times (i.e., each time randomly assigning patients and controls labels to the 38 individuals, 19 controls and 19 depressed patients) and repeated the cross-validation procedure. We then counted the number of times the “True Positive” and the “True Negative” ratios for the permuted labels were higher than the ones obtained for the real labels. Dividing this number by 1000 allowed us to compute a p-value.

## Results

### Patient classification as an outlier detection problem

**Fig. 2:**
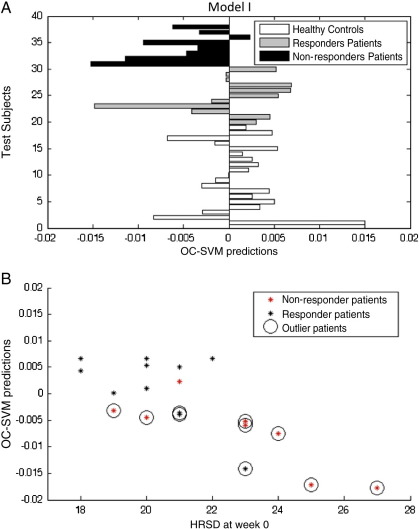
(A) Test predictions for the OC-SVM trained with the healthy controls using voxel-based features. If the test prediction is below zero, the subject is considered an outlier in relation to the training group. (B) Scatter plot between the OC-SVM patients' predictions and the HRSD at the time of the scan (week 0).

**Fig. 3:**
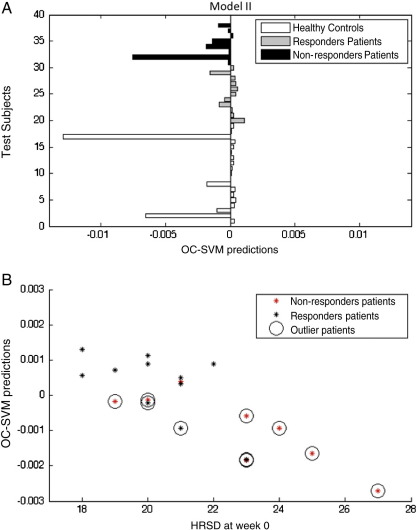
(A) Test predictions for the OC-SVM trained with the healthy controls using region-based features. If the test prediction is below zero, the subject is considered an outlier in relation to the training group. (B) Scatter plot between the OC-SVM patients' predictions and the HRSD at the time of the scan (week 0).

In Tables 2 and 3 and Figs. 2 and 3 we present the results of the OC-SVMs considering healthy controls as the positive class and using whole brain voxel-based and region-based features, respectively.

True Positive (TP) corresponds to the percentage of negative examples (i.e. depressed patients) detected as outliers by the classifier. The True Negative (TN) corresponds to the percentage of positive examples (i.e. healthy controls) detected as non-outliers by the classifier.

The OC-SVM applied to voxel-based features detected 63% of healthy controls subjects as non-outliers and 63% of the patients as outliers. When applied to region based features the OC-SVM detected 79% of healthy controls as non-outliers and only 52% of the patients as outliers. However in both cases, most of the patients' classifiers as non-outliers responded to treatment (85% in the first model and 89% in the second model) and most of the patients classified as outliers did not respond to treatment (58% in the first model and 70% in the second model). These results show that the OC-SVM was able to identify two subgroups within the patients whose membership was associated with the response to treatment. In both models patients classified as non-outliers have much higher probability to respond to treatment in relation to the ones classified as outliers.

We found a significant correlation (Pearson) between the test predictions of all patients (i.e. the distance from the test examples to the decision boundary) and the Hamilton Rating Scale for Depression (HRSD) (Figs. 2B and 3B). In both cases the patients' predictions were generated using data from all controls to train the OC-SVMs and using data from all patients for testing. Therefore, for each condition the test predictions for all patients were computed in relation to the same classifier and could be compared. We also observed that, in both models, the correlation between the test prediction and the HRSD score was higher for the patients that did not respond to treatment than for those that did respond to treatment, which provides additional evidence that the patient population is heterogeneous with at least two sub-groups.

The fact that the test predictions were correlated with the Hamilton Rating Scale for Depression is an important validation that the OC-SVM is finding a meaningful boundary of the distribution of brain activation patterns in healthy controls (i.e. the more severe is the depression the greater is the departure from the “normality boundary”).

Tables 4 and 5 show the results when the OC-SVM was trained with patients as a positive class. When using voxel-based features 31% of the controls and 37% of the patients were detected as outliers and when using region-based features 15% of the controls and 32% of the patients were detected as outliers. These results are also evidence that the patient group is more heterogeneous than the control group and therefore the hypersphere or decision boundary that encloses most of the patients contains data in the healthy control range.

### OC-SVM maps

**Fig. 4:**
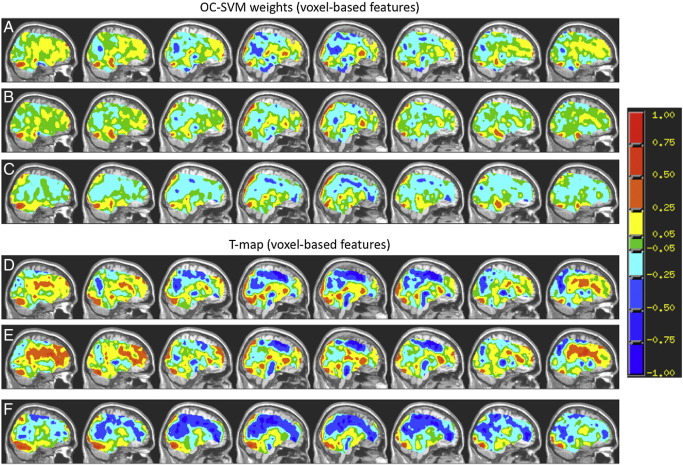
Pre-image approximation for the OC-SVM weights using voxel-based features (for low A, medium B and high C intensity of sadness, respectively) and statistical maps based on the positive class (for low D, medium E and high F intensity of sadness, respectively). In both cases the maps were overlaid onto an anatomical template using AFNI (http://afni.nimh.nih.gov/afni). To generate a color bar that is symmetric around zero, the values in the maps are rescaled in such a way that the absolute maximum is assigned the value of + 1 and the color scale runs from − 1 to + 1. The red areas indicate positive weights in the decision function and the blue areas indicate negative weights in the decision function.

**Fig. 5:**
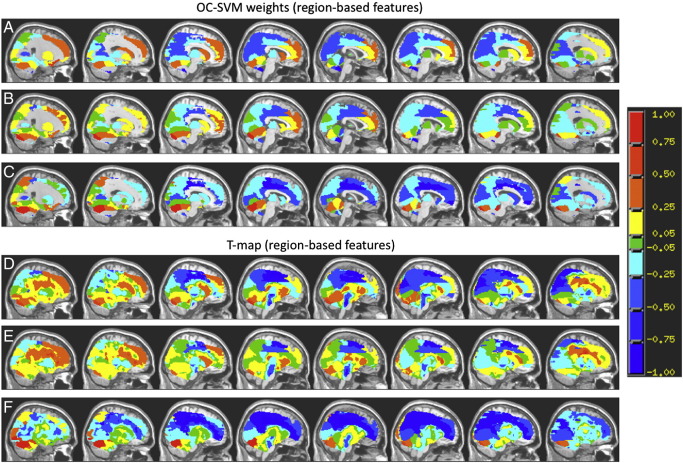
Pre-image approximation for the OC-SVM weights and statistical maps based on the positive class using region-based features (the images were created using the same procedure described in Fig. 4).

In Figs. 4(A–C) and 5(A–C) we present the maps corresponding to the weights or pre-image approximations for the OC-SVM trained with healthy controls for the models using voxel-based and region-based features, respectively. For comparative purposes we also present the statistical maps (unthresholded) based on the positive class computed applying one-sample t-tests to the same data used as input to the OC-SVM (Figs. 4D–F and 5D–F). The OC-SVM weight determines the centre of a single Gaussian capturing the support vector examples (Schölkopf et al., 1999) therefore it defines the boundaries of the “hypersphere”. It is important to emphasize that as for other pattern recognition approaches the OC-SVM is a multivariate technique based on the whole pattern therefore one cannot make local inferences about the discriminating regions. In our framework we are combining the patterns of brain response for the three intensities of the emotional expression (low, medium and high intensity of sadness) into a single vector consequently the OC-SVM explored the information contained in the three patterns.

In Figs. 4 and 5 we present the OC-SVM weights and T-maps (for each intensity of the emotional expression) using voxel-based and region-based features, respectively. As we can see from Figs. 4 and 5 there is a good agreement between the distributions of positive and negative between the two approaches, voxels and region-based features. In Tables S1 A–B (Supplementary Material) we list the top 15 clusters with the highest weights and the top 15 clusters with the highest t-values. Using voxel-based features produces one weight value per voxel. In Tables S2 A–B (Supplementary Material) we listed the top 15 regions with highest weights and the top 15 regions with highest t-values. Using region-based features produces one weight per region.

Although there are some similarities between the OC-SVM maps and the T-maps for the same emotional expression we can also see many differences among them (which can be observed in Tables S1–S2, in the Supplementary Material, describing the top clusters for both approaches). It is important to emphasize that these two approaches are conceptually different, the OC-SVM map represents a multivariate decision boundary and it shows the relative weight of the features (voxels or regions) to the decision function, i.e. voxels with higher weights will contribute more for the decision if a new example is an outlier or not. On the other hand the T-map shows local effects, i.e. the value in each feature (voxel or region) corresponds to a univariate statistical test that test if the mean of the positive class is “significantly different” from zero given the dispersion (standard error) of the sample. From Figs. 4 and 5 we can see that the decision (made by the OC-SVM with RBF kernel) of classifying an example as an outlier in relation to a specific class was not only driven by the local effects (if this was the case the OC-SVM and the T-maps would be very similar). This is evidence that more complex relations between the features (e.g. correlations, etc.) also play an important role in this decision.

We would like to emphasize that due to the non-linearity of the RBF kernel, positive weights are a non-linear function of activation and negative weights are a non-linear function of deactivation and therefore the maps should not be interpreted as a simple mean pattern among the group. The OC-SVM weight map is a spatial representation of the decision function, i.e. the value of the voxel corresponds to its weight or contribution to the decision function (i.e. to the classification). Voxels with positive values will contribute to classifying the subject as non-outlier and voxels with negative values will contribute to classifying the subject as outlier.

## Discussion

In the present work we propose an application of the OC-SVM as a framework to assess deviations from the boundary of a specific class or population based on neuroimaging data. As a proof of concept, we use this framework to investigate whether patterns of fMRI response to sad facial expressions in depressed patients would be classified as outliers in relation to a boundary defined based on patterns of healthy control subjects. We showed that a patient's deviation from the “healthy control” boundary was correlated with their Hamilton Rating Scale for Depression scores. In addition, the OC-SVM was able to identify two subgroups within the patients whose group memberships were associated with their responses to treatment.

These results were replicated using two different approaches to define features: whole brain voxels and whole brain regions. Although the two approaches create feature vectors with very different numbers of dimensions (517,647 in the first case and 348 in the second case) the main results were remarkably similar. Our results illustrate the potential of using pattern recognition approaches to derive quantitative measures of brain abnormality based on neuroimaging data that quantify the degree of abnormality in brain anatomy or function caused by a psychiatric or neurological disorder. The development of diagnostic and prognostic biomarkers for psychiatric disorders based on neuroimaging data requires the availability of techniques that can identify individual functional and/or anatomical alterations in the brain due to the illness and can use this information to assign group membership for new subjects.

Pattern recognition approaches, such as Support Vector Machine (SVM) learning, have been used to classify patterns of brain activity elicited by sensory or cognitive processes as ‘mind-reading’ devices that can predict an individual's brain state (e.g. Mourão-Miranda et al., 2005; Haynes and Rees, 2006; Norman et al., 2006). In a clinical context, these approaches have mainly been applied to classify groups of individuals based on brain structural Magnetic Resonance Imaging (MRI) data (e.g. Fan et al., 2008; Soriano-Mas et al., 2007; Klöppel et al., 2008; Koutsouleris et al., 2009). Only a few studies have applied similar methods to functional Magnetic Resonance Imaging (fMRI) data (e.g. Fu et al., 2008; Marquand et al., 2008). These latter studies reported that healthy controls and unipolar depressed patients could be discriminated by whole brain patterns of activation to a specific stimulus (sad faces, Fu et al., 2008) or task (verbal working memory, Marquand et al., 2008). However, none of these studies has addressed the more general question of defining a boundary characterizing a distribution of patterns of brain activation or anatomy of a normal population, and in relation to which patterns of patients would be classified as outliers. The latter question consists of a “one-class classification problem”, where, instead of learning how to discriminate between two classes, one is interested in defining properties of a specific class and identifying outlier examples. This framework can be very useful in situations where sufficient data from one class are not available for training a standard two-class classifier or one is interested in finding outliers within a specific population. If there are only a small number of patient data available the standard two class approach is problematic, as training with extremely unbalanced group sizes might lead to a significant bias in the classification (e.g. a classifier trained with 80% of examples of class 1 and 20% of examples of class 2 can get 80% accuracy by always guessing class 1). In those situations one can still train the OC-SVM with the healthy control group and potentially detect the patients as outliers. The one-class learning approach is also advantageous in cases where the clinical diagnosis is unclear or there are subgroups in the patient population, i.e. it would be counterproductive to give the same label for all the patients using a standard two class learning approach. Another possible application of the OC-SVM approach could be multi-class problems where one OC-SVM would be trained for each class and new examples would be tested against each of classifiers in order to find the one with the least deviation. One could then use the confusion matrix based on multiple OC-SVMs to measure overlap between different classes.

The OC-SVM has been previously applied to connectivity networks of healthy subjects (Sato et al., 2009) and to fMRI data to learn the pattern of activity associated with a motor task (Hardoon and Manevitz, 2005). From a technical point of view the novelty of the current work in relation to the previous work using OC-SVM is the application to whole brain volumes and region-based features and the generation of the OC-SVM maps using pre-image approximation (OC-SVM maps). From an application point of view, the novelty is to use the OC-SVM framework to treat patient classification as an outlier detection problem.

In contrast to two-class pattern classification approaches, the OC-SVM can quantify the deviation of a single person's pattern of brain activation from the boundary of the distribution of positive patterns (e.g. deviations from the “healthy control” boundary). As shown in this study, the amount of deviation from the “healthy control” boundary is correlated with the severity of symptoms. From a clinical perspective, OC-SVM thus might allow a quantification of symptom severity based solely on neuroimaging data. By analogy with psychometric test construction, a cut-off value is determined by locating a subject's test value in relation to the distribution derived from a number of control subjects. The fact that the OC-SVM approach can rely entirely on data from a control sample makes it particularly suitable for the identification of rare disorders when only data from very small numbers of patients are available. In addition it may even find clinical utility when benchmarking individual patients to a normative database. Another possible application of the OC-SVM is as a clustering approach. In this case the algorithm is trained to find the “most outlying examples” in relation to the rest of the group. The percentage of outliers is defined by the parameter ν, which can be defined a priori.

Perhaps more interesting is the fact that if we split the patients in two groups (i.e. responders and non-responders to a treatment) in both models, most of the patients classified as non-outliers responded to treatment (85% in the first model and 89% in the second model) and most of the patients classified as outliers did not respond to treatment (58% in the first model and 70% in the second model). In addition the correlation of the OC-SVM prediction with HDRS scores (i.e. standardized external expert-ratings of symptom severity) also predicted treatment response. Specifically, patients who did not respond to treatment showed higher correlation between the HDRS and the OC-SVM predictions. In contrast, treatment was more successful in patients with incongruent symptom severity estimates (Tables 2 and 3). One possible explanation for these results is that patients who responded to treatment might have reported an HRSD that is higher than their ‘brain level of depression’ as indicated by fMRI. Predicting future response to treatment based on fMRI is an extremely challenging problem, which has economical and clinical implications. Further studies with larger sample size are necessary to confirm the potential use of OC-SVM to predict future response to treatment.

It should be emphasized that in the present work the clinical information from the patients was not used to train the OC-SVM algorithm therefore finding correlations between the patients' predictions and the HRSD is an important indication that the algorithm can find a meaningful boundary characterizing the distribution of patterns of brain activation in response to sad facial expressions in the healthy control group. These results show that the pattern of fMRI response to sad faces in healthy subjects is homogeneous enough to enable the definition of a “normality boundary”, the amount of departure from this boundary is correlated to the severity of depression and this correlation is higher for patients who did not responded to treatment.

The areas with higher values in the OC-SVM weights and T-maps (Figs. 4 and 5 and Tables S1 and S2) are in agreement with areas that were identified in previous studies as having different activation patterns between healthy controls and depressed patients. Specifically, a highly similar pattern of discriminating regions for low intensity of sadness including medial frontal gyrus, the cuneus, and parietal regions has been identified multiple times using pattern recognition methods (Fu et al., 2008; Hahn et al., 2011). Furthermore, the involvement of clusters within the caudate as well as in frontal regions is in line with previous evidence from task-related functional (Fu et al., 2008; Keedwell et al., 2005; Knutson et al., 2008; Epstein et al., 2006; Pizzagalli et al., 2009) as well as structural and metabolic data (Krishnan et al., 1992; Bremner et al., 2002; Ito et al., 1996; Kennedy et al., 1997) in depression. As similar discriminative patterns including regions also relevant in this study have been observed over a variety of tasks, more general metabolic alterations might be relevant: especially in the occipital and frontal regions in question, differences in GABA and glutamate levels between depressive patients and controls have been observed (Sanacora et al., 1999; Sanacora et al., 2004; Hasler et al., 2007). This might impact glutamatergic neurotransmission directly as well as indirectly via structural changes (loss of tissue; Bhagwagar et al., 2008). Considering the central role of astrocytes for both GABAergic neurotransmission (through the GABA precursor glutamine) and the BOLD response (through neurovascular coupling; Rossi, 2006), these metabolic alterations in depressive patients might impact measurements during all paradigms and conditions. Although beyond the scope of this work, it would be highly interesting to examine the interaction of (persistent) metabolic changes and (dynamic) task-related alterations in depression and their impact on classification.

One drawback of the one-class approach is that it does not provide specificity for specific disorders (as different psychiatric illnesses might affect the brain functions in similar ways) but is a first step towards deriving a measure of “abnormality” or “atypicality” via brain imaging. The same technical framework could then be used in a second stage to define patterns of brain activity specific to different disorders. Another limitation of our study is the small sample size. The present work is nevertheless a proof of concept that the one class framework can be applied to neuroimaging to find outliers (negative class) in relation to a positive class used for training. A future direction is to apply this framework to larger samples of healthy control subject and different patient groups.

## Figures and Tables

**Table 1:** Demographic features.

	Depressed patients	Healthy controls
N = 19	N = 19
Mean age (years)	43.2 (8.8)	42.8 (6.7)
Gender (m/f)	6/13	8/11
Full IQ	109.2 (14.5)	116.4 (18.8)
HRSD at week 0	21.1 (2.3)	0.3 (0.7)
HRSD at week 8	8.5 (4.8)	0.0 (0.0)

*Responders*		
Patients with final HRSD ≤ 10 (m/f)	2/6	
Mean age	44.9 (10.8)	
HRSD at week 0	20.1 (1.8)	
HRSD at week 8	4.2 (1.6)	

*Non-responders*		
Patients with final HRSD > 10 (m/f)	4/7	
Mean age	43.3 (7.4)	
HRSD at week 0	21.9 (2.7)	
HRSD at week 8	12.9 (4.8)	

Mean scores for each variable are presented with standard deviations in parentheses; HRSD = Hamilton Rating Scale for Depression.

**Table 2:** Model I.

Positive class	Healthy controls	
Features	Whole brain voxels	
Kernel	RBF	
Model parameters (search range)	ν	0.1–0.5
Sigma	1e−07–1e−03
True Negative (% Controls detected as non-outlier)	63% (p-value = 0.048)^a^	
True Positive (% Patients detected as outlier)	63%(p-value = 0.048)^a^	
% Patients outliers that responded to treatment	42%	
% Patients non-outliers that responded to treatment	85%	
Number of Support Vectors	6	
Correlation between HRSD and OCSVM predictions: all patients	− 0.78 (p = 6e−5)	
Correlation between HRSD and OCSVM predictions: patients responders	− 0.83 (p = 0.01)	
Correlation between HRSD and OCSVM predictions: patients non-responders	− 0.57 (p = 0.06)	

a Determined by permutation test.

**Table 3:** Model II.

Positive class	Healthy controls	
Features	Whole brain regions	
Kernel	RBF	
Model parameters (search range)	ν	0.1–0.5
Gamma	1e−07–1e−03
True Negative (% controls detected as non-outlier)	79% (p-value = 0.01)^a^	
True Positive (% patients detected as outlier)	52% (p-value = 0.01)^a^	
% Patients outliers that responded to treatment	30%	
% Patients non-outliers that responded to treatment	89%	
Number of Support Vectors	4	
Correlation between HRSD and OCSVM predictions: patients	− 0.81 (p = 2.2e−5)	
Correlation between HRSD and OCSVM predictions: patients responders	− 0.86 (p = 0.006)	
Correlation between HRSD and OCSVM predictions: patients non-responders	− 0.61 (p = 0.04)	

a Determined by permutation test.

**Table 4:** Model III.

Positive class	Patients	
Features	Whole brain voxels	
Kernel	RBF	
Model parameters (search range)	ν	0.1–0.5
Sigma	1e−07–1e−03
% Patients detected as non-outlier	63%	
% Controls detected as outlier	31%	
Number of Support Vectors	7	

**Table 5:** Model IV.

Positive class	Patients	
Features	Whole brain regions	
Kernel	RBF	
Model parameters (search range)	ν	0.1–0.5
Sigma	1e−07–1e−03
% Patients detected as non-outlier	68%	
% Controls detected as outlier	15%	
Number of Support Vectors	4	

## Appendix A OC-SVM formulation

Here we present a brief description of the OC-SVM formulation, but we refer the reader to other sources for a more detailed introduction (Schölkopf et al., 2001; Shawe-Taylor and Cristianini, 2004).

Considering a training data

(1) $$X=\{x_{1},\ldots ,x_{m}\}\subset \chi$$

where *m* is the number of observation and *χ* is a compact subset of $R^{N}$ (input space). Let *ϕ* be a feature map χ→I, that is, a map into an inner product space (feature space) I such that the dot product in this feature space can be computed by evaluating a simple kernel

(2) $$k(x,x^{\prime })=(\phi (x).\phi (x^{\prime }))$$

such as the Gaussian

(3) $$k(x,x^{\prime })=e^{-||x-x^{\prime }|{|}^{2}/2s^{2}}$$

where *s* is the width of the Gaussian kernel.

The data can be separated from the origin by solving the following quadratic program:

(4) $$\begin{array}{l} \min\frac{1}{2}||w|{|}^{2}+\frac{1}{\nu m}\sum_{i}\xi_{i}-\rho \\ \text{subject to}(w\text{.}\phi (x_{i}))\geq \rho -\xi_{i},\xi_{i}\geq 0 \end{array}$$

where *ν* ∈ (0, 1] is a parameter that characterizes the fractions of outliers (i.e. proportion of data points for which the function *f* that takes the value − 1) and *ξ*_*i*_ are the slack variables. **w** and *ρ* can be found by solving the optimization problem (4), then the decision function has the form

(5) f(x)=sgn((w.ϕ(x))−ρ)

and will equal 1 for most examples **x**_i_ contained in the training set and − 1 for the others (i.e. the outliers). By introducing Lagrangian multipliers *α*_*i*_, *β*_*i*_ ≥ 0, one obtains

(6) $$\begin{array}{l} L(w,\xi ,\rho ,\alpha ,\beta )=\frac{1}{2}||w|{|}^{2}+\frac{1}{\nu m}\sum_{i}\xi_{i}-\sum_{i}\beta_{i}\xi_{i}-\rho \\ -\sum_{i}\alpha_{i}((w\text{.}\phi (x_{i}))-\rho +\xi_{i})\text{.} \end{array}$$

Setting the derivatives with respect to the primal variables w,ξ,ρ to zero yields

(7) $$w=\sum_{i}\alpha_{i}\phi (x_{i})$$

(8) $$\alpha_{i}=\frac{1}{\nu m}-\beta_{i}\leq \frac{1}{\nu m}$$

(9) $$\sum_{i}\alpha_{i}=1\text{.}$$

Hence the decision function has the form

(10) $$f(x)=\mathrm{sgn}(\sum_{i}\alpha_{i}k(x_{i},x)-\rho )\text{.}$$

The multipliers *α*_*i*_ can be solved from the dual problem:

(11) $$\begin{array}{l} \min\frac{1}{2}\sum_{ij}\alpha_{i}\alpha_{j}k(x_{i},x_{j})\\ \text{s}.\text{t}.0\leq \alpha_{i}\leq \frac{1}{\nu m},\sum_{i}\alpha_{i}=1\text{.} \end{array}$$

The parameter *ρ* can be recovered by exploiting that for any such *α*_*i*_, the corresponding pattern $x_{i}$ satisfies

(12) $$\rho =(w\text{.}\phi (x_{i}))=\sum_{j}\alpha_{j}k(x_{j},x_{i})$$

## Appendix B Pre-image approximation for RBF kernel

This section is a summary of the pre-image approximation algorithm proposed by Schölkopf et al. (1999). Non-linear kernel approaches, such as the OC-SVM described in Appendix A, express their solutions as a linear combination of mapped input points (e.g. Eq. (7)). However, as the map *ϕ* into the feature space I is nonlinear one cannot generally assert that such solution will have a pre-image under *ϕ*, i.e. a point $z\in R^{N}$ such that ϕ(z)=w (not each point in the span of the mapped input data is necessarily the image of some input pattern). If there exists a $z\in R^{N}$ such that ϕ(z)=w and if *k* is an invertible function *f*_*k*_ of $\text{(}x_{j}\text{. }x_{i})$, then z can be computed as

(13) $$z=\sum_{i=1}^{N}f_{k}{}^{-1}\left(\sum_{j=1}^{m}\alpha_{j}k(x_{j},e_{i})\right)e_{i}$$

where {*e*_1_, …, *e*_*N*_} is any orthonormal basis of the input space.

Unfortunately there are many kernels such as the RBF or Gaussian kernel for which there are no pre-images. In these cases one can find approximate pre-images. $z\in R^{N}$ is an approximate pre-image for w if

(14) $$\rho (z)={\left\|w-\phi (z)\right\|}^{2}$$

is small.

In practice instead of minimizing Eq. (14) one can minimize the distance between w and the orthogonal projection of w onto the span ϕ(z) Eq. (15), which comprises a lower dimensional problem

(15) $${\left\|\frac{\left(w.\phi (z)\right)}{\left(\phi (z).\phi (z)\right)}\phi (z)-w\right\|}^{2}={\left\|w\right\|}^{2}-\left(\frac{{\left(w.\phi (z)\right)}^{2}}{\left(\phi (z).\phi (z)\right)}\right)\text{.}$$

This corresponds to maximizing

(16) $$\frac{{\left(w.\phi (z)\right)}^{2}}{\left(\phi (z).\phi (z)\right)}$$

which can be expressed in terms of the kernel.

Here we present a previously proposed fixed point iteration method to solve this problem. For Kernels which satisfy k(z,z) = 1 for all $z\in R^{N}$ (e.g. RBF kernel), Eq. (16) reduces to

(17) $${\left(w.\phi (z)\right)}^{2}\text{.}$$

Setting the gradient of Eq. (17) to zero one obtains

(18) $$0=\nabla_{z}{\left(w.\phi (z)\right)}^{2}=2(w.\phi (z))\nabla_{z}(w.\phi (z))\text{.}$$

To evaluate the gradient in terms of kernels Eq. (7) is substituted

(19) $$0=\sum_{i=1}^{Nsv}\alpha_{i}\nabla_{z}(\phi (x_{i}).\phi (z))=\sum_{i=1}^{Nsv}\alpha_{i}\nabla_{z}k(x_{i},z)\text{.}$$

Where *Nsv* is the number of support vectors.

For $k(x_{i},z)=k\left({\left\|x_{i}-z\right\|}^{2}\right)$ one obtains

(20) $$z=\frac{\sum_{i=1}^{Nsv}\alpha_{i}k'\left({\left\|x_{i}-z\right\|}^{2}\right)x_{i}}{\sum_{i=1}^{Nsv}\alpha_{i}k'\left({\left\|x_{i}-z\right\|}^{2}\right)}\text{.}$$

For the Gaussian kernel $k(x,z)=e^{-||x-z|{|}^{2}/2s^{2}}$ one obtains

(21) $$z=\frac{\sum_{i=1}^{Nsv}\alpha_{i}e^{-||x_{i}-z|{|}^{2}/2s^{2}}x_{i}}{\sum_{i=1}^{Nsv}\alpha_{i}e^{-||x_{i}-z|{|}^{2}/2s^{2}}}$$

which leads to the iterative scheme

(22) $$z_{t+1}=\frac{\sum_{i=1}^{Nsv}\alpha_{i}e^{-||x_{i}-z_{t}|{|}^{2}/2s^{2}}x_{i}}{\sum_{i=1}^{Nsv}\alpha_{i}e^{-||x_{i}-z_{t}|{|}^{2}/2s^{2}}}\text{.}$$

The algorithm stops if the difference between the old and new value of z is small then a certain value or if a maximum number of iterations is achieved.

In the present work z was plotted as a brain image and it corresponds to a pre-image approximation of the RBF weight vector in the input space.

## Appendix C One-sample t-tests

The t-maps were generated using the following equation

(23) ti=x¯i−μsin

where ${\bar{x}}_{i}$ and *s*_*i*_ are the sample mean and standard deviation of the beta values for the voxel *i*, *n* is the sample size (i.e. number of subjects) and *μ* is equal to zero.

## References

[bb0005] Bhagwagar Z., Wylezinska M., Jezzard P., Evans J., Boorman E., M Matthews P., J Cowen P. (2008). Low GABA concentrations in occipital cortex and anterior cingulate cortex in medication-free, recovered depressed patients. Int. J. Neuropsychopharmacol..

[bb0010] Bremner J.D., Vythilingam M., Vermetten E., Nazeer A., Adil J., Khan S., Staib L.H., Charney D.S. (2002). Reduced volume of orbitofrontal cortex in major depression. Biol. Psychiatry.

[bb0015] Ekman P., Friesen W. (1976). Pictures of Facial Affect.

[bb0020] Epstein J., Pan H., Kocsis J.H., Yang Y., Butler T., Chusid J., Hochberg H., Murrough J., Strohmayer E., Stern E., Silbersweig D.A. (2006). Lack of ventral striatal response to positive stimuli in depressed versus normal subjects. Am. J. Psychiatry.

[bb0025] Fan Y., Batmanghelich N., Clark C., Davatzikos C. (2008). Spatial patterns of brain atrophy in MCI patients, identified via high-dimensional pattern classification, predict subsequent cognitive decline. NeuroImage.

[bb0030] First M., Spitzer R., Gibbon M., Williams J. (1995). Structured Clinical Interview for DSM-IV Axis I Disorders (SCID-I).

[bb0035] Fu C., Williams S., Cleare A. (2004). Attenuation of the neural response to sad faces in major depression by antidepressant treatment: a prospective, event-related functional magnetic resonance imaging study. Arch. Gen. Psychiatry.

[bb0040] Fu C.H., Mourao-Miranda J., Costafreda S.G., Khanna A., Marquand A.F., Williams S.C., Brammer M.J. (2008). Pattern classification of sad facial processing: toward the development of neurobiological markers in depression. Biol. Psychiatry.

[bb0050] Hahn, T., Marquand, A.F., Ehlis, A.C., Dresler, T., Kittel-Schneider, S., Jarczok, T.A., Lesch, K.P., Jakob, P.M., Mourao-Miranda, J., Brammer, M.J., Fallgatter, A.J., 2011. Integrating Neurobiological Markers of Depression. Arch. Gen. Psychiatry 68 (4), 361–368 (May). 10.1001/archgenpsychiatry.2010.178 21135315

[bb0055] Hamilton M. (1960). A rating scale for depression. J. Neurol. Neurosurg. Psychiatry.

[bb0060] Hardoon D., Manevitz L. (2005). fMRI analysis via one-class machine learning techniques. Paper presented at: In Proceedings of the 19th International Joint Conference on Artificial Intelligence (IJCAI).

[bb0065] Hasler G., van der Veen J.W., Tumonis T., Meyers N., Shen J., Drevets W.C. (2007). Reduced prefrontal glutamate/glutamine and gamma-aminobutyric acid levels in major depression determined using proton magnetic resonance spectroscopy. Arch. Gen. Psychiatry.

[bb0070] Haynes J., Rees G. (2006). Decoding mental states from brain activity in humans. Nat. Rev. Neurosci..

[bb0075] Ito H., Kawashima R., Awata S., Ono S., Sato K., Goto R., Koyama M., Sato M., Fukuda H. (1996). Hypoperfusion in the limbic system and prefrontal cortex in depression: SPECT with anatomic standardization technique. J. Nucl. Med..

[bb0080] Keedwell P.A., Andrew C., Williams S.C., Brammer M.J., Phillips M.L. (2005). The neural correlates of anhedonia in major depressive disorder. Biol. Psychiatry.

[bb0085] Kennedy S.H., Javanmard M., Vaccarino F.J. (1997). A review of functional neuroimaging in mood disorders: positron emission tomography and depression. Can. J. Psychiatry.

[bb0090] Kjems U., Hansen L., Anderson J. (2002). The quantitative evaluation of functional neuroimaging experiments: mutual information learning curves. NeuroImage.

[bb0095] Klöppel S., Stonnington C., Chu C. (2008). Automatic classification of MR scans in Alzheimer's disease. Brain.

[bb0100] Knutson B., Bhanji J.P., Cooney R.E., Atlas L.Y., Gotlib I.H. (2008). Neural responses to monetary incentives in major depression. Biol. Psychiatry.

[bb0105] Koutsouleris N., Meisenzahl E., Davatzikos C. (2009). Use of neuroanatomical pattern classification to identify subjects in at-risk mental states of psychosis and predict disease transition. Arch. Gen. Psychiatry.

[bb0110] Krishnan K.R., McDonald W.M., Escalona P.R., Doraiswamy P.M., Na C., Husain M.M., Figiel G.S., Boyko O.B., Ellinwood E.H., Nemeroff C.B. (1992). Magnetic resonance imaging of the caudate nuclei in depression. Preliminary observations. Arch. Gen. Psychiatry.

[bb0115] Lao Z., Shen D., Xue Z., Karacali B., Resnick S., Davatzikos C. (2004). Morphological classification of brains via high-dimensional shape transformations and machine learning methods. NeuroImage.

[bb0120] Marquand A., Mourão-Miranda J., Brammer M., Cleare A., Fu C. (2008). Neuroanatomy of verbal working memory as a diagnostic biomarker for depression. Neuroreport.

[bb0125] Mitrushina M., Boone K., D'Elia L. (1999). Handbook of Normative Data for Neuropsychological Assessment.

[bb0130] Mourão-Miranda J., Bokde A., Born C., Hampel H., Stetter M. (2005). Classifying brain states and determining the discriminating activation patterns: Support Vector Machine on functional MRI data. NeuroImage.

[bb0135] Norman K., Polyn S., Detre G., Haxby J. (2006). Beyond mind-reading: multi-voxel pattern analysis of fMRI data. Trends Cogn. Sci..

[bb0140] Pizzagalli D.A., Holmes A.J., Dillon D.G., Goetz E.L., Birk J.L., Bogdan R., Dougherty D.D., Iosifescu D.V., Rauch S.L., Fava M. (2009). Reduced caudate and nucleus accumbens response to rewards in unmedicated individuals with major depressive disorder. Am. J. Psychiatry.

[bb0145] Rossi D.J. (2006). Another BOLD role for astrocytes: coupling blood flow to neural activity. Nat. Neurosci..

[bb0150] Sanacora G., Mason G.F., Rothman D.L., Behar K.L., Hyder F., Petroff O.A., Berman R.M., Charney D.S., Krystal J.H. (1999). Reduced cortical gamma-aminobutyric acid levels in depressed patients determined by proton magnetic resonance spectroscopy. Arch. Gen. Psychiatry.

[bb0155] Sanacora G., Gueorguieva R., Epperson C.N., Wu Y.T., Appel M., Rothman D.L., Krystal J.H., Mason G.F. (2004). Subtype-specific alterations of gamma-aminobutyric acid and glutamate in patients with major depression. Arch. Gen. Psychiatry.

[bb0160] Sato, J.R., da Graça Morais Martin, M., Fujita, A., Mourão-Miranda, J., Brammer, M.J., Amaro Jr., E., 2009. An fMRI normative database for connectivity networks using one-class support vector machines. Hum. Brain Mapp. 30 (4), 1068–1076 (Apr). 10.1002/hbm.20569 PMC6870648 18412113

[bb0200] Schölkopf, B., Smola, A., 2002. Learning with Kernels. MIT Press.

[bb0165] Schölkopf B., Mika S., Burges C.J.C., Knirsch P., Müller K.R., Rätsch G., Smola A.J. (1999). Input space versus feature space in kernel-based methods. IEEE Trans. Neural Netw..

[bb0170] Schölkopf B., Platt J., Shawe-Taylor J., Smola A., RCW (2001). Estimating the support of a high-dimensional distribution. Neural Comput..

[bb0175] Shawe-Taylor J., Cristianini N. (2004). Kernel Methods for Pattern Analysis.

[bb0180] Soriano-Mas C., Pujol J., Alonso P. (2007). Identifying patients with obsessive-compulsive disorder using whole-brain anatomy. NeuroImage.

[bb0185] Talairach J., Tournoux P. (1988). Co-planar Stereotaxic Atlas of the Human Brain : 3-Dimensional Proportional System; an Approach to Cerebral Imaging.

[bb0190] Tzourio-Mazoyer N., Landeau B., Papathanassiou D., Crivello F., Etard O., Delcroix N., Mazoyer B., Joliot M. (2002). Automated anatomical labeling of activations in SPM using a macroscopic anatomical parcellation of the MNI MRI single-subject brain. NeuroImage.

[bb0195] Vapnik V.N. (1995). The Nature of Statistical Learning Theory.

