# Supplementary material for: Patient classification as an outlier detection problem: An application of the One-Class Support Vector Machine
Source: Neuroimage. 2011 Oct 1;58(3-4):793–804. doi: 10.1016/j.neuroimage.2011.06.042 (PMC3191277; doi:10.1016/j.neuroimage.2011.06.042)
Supplement: Supplementary file 1 — Supplementary materials. [file mmc1.doc]

**Supplementary Material:** Tables describing peaks of the OC-SVM weights and T-maps

In Tables S1A-B the clusters were selected using 3Dclust in AFNI (<http://afni.nimh.nih.gov/afni>) and the anatomical regions were estimated using the software Talairach Client (<http://www.talairach.org/>) and the coordinates of the clusters’ peaks.

Table S1A. Top clusters of the OC-SVM maps using voxel-based features

| **Low intensity of sadness** | | | | | |
| --- | --- | --- | --- | --- | --- |
| **Volume** | **Max Int** | **X coor** | **Y coor** | **Z coor** | **Region** |
| 140 | 9.4019 | 4 | -96 | 22 | Cuneus |
| 1255 | 6.9045 | -52 | -58 | -22 | Declive |
| 312 | 6.1956 | -46 | 0 | -22 | Middle Temporal Gyrus |
| 732 | -4.9491 | 0 | -54 | 20 | Posterior Cingulate |
| 526 | -4.825 | 10 | -16 | -26 | No Gray Matter found |
| 127 | 4.7003 | -50 | -46 | 54 | Inferior Parietal Lobule |
| 200 | 4.5786 | 44 | 0 | -26 | Middle Temporal Gyrus |
| 156 | 4.4668 | 18 | -34 | -12 | Culmen |
| 123 | 4.4589 | 66 | -42 | -4 | Middle Temporal Gyrus |
| 164 | 4.4167 | 56 | 18 | -6 | Inferior Frontal Gyrus |
| 52 | 4.1736 | -12 | -38 | -16 | Culmen |
| 78 | -3.9852 | -2 | -32 | -8 | Culmen |
| 133 | -3.964 | -10 | -78 | 26 | Cuneus |
| 92 | 3.8098 | 2 | 16 | 8 | Caudate |
| 56 | -3.7806 | 2 | -6 | 62 | Medial Frontal Gyrus |
|  | | | | | |
| **Medium intensity of sadness** | | | | | |
| **Volume** | **Max Int** | **X coor** | **Y coor** | **Z coor** | **Regions** |
| 692 | 12.41 | 4 | -80 | 52 | Precuneus |
| 1884 | 8.8854 | -50 | -58 | -24 | Tuber |
| 321 | 7.3434 | -46 | -48 | 58 | Inferior Parietal Lobule |
| 337 | 7.0488 | -16 | -26 | -24 | Culmen |
| 351 | 6.5235 | 18 | -34 | -22 | Culmen |
| 262 | 6.2792 | 30 | 64 | 6 | Middle Frontal Gyrus |
| 727 | 5.8895 | 54 | 20 | -8 | Inferior Frontal Gyrus |
| 248 | 5.7513 | -46 | 0 | -22 | Middle Temporal Gyrus |
| 161 | 5.488 | 48 | -50 | 58 | Inferior Parietal Lobule |
| 262 | -5.3923 | 0 | -56 | 18 | Posterior Cingulate |
| 229 | 4.978 | 0 | 20 | 10 | Caudate |
| 141 | 4.9454 | 66 | -42 | -2 | Middle Temporal Gyrus |
| 544 | -4.7988 | -40 | -24 | 54 | Postcentral Gyrus |
| 96 | -4.7462 | 0 | -34 | -8 | Culmen |
|  | | | | | |
| **High intensity of sadness** | | | | | |
| **Volume** | **Max Int** | **X coor** | **Y coor** | **Z coor** | **Regions** |
| 1206 | 11.715 | 4 | -80 | 52 | Precuneus |
| 4448 | 10.385 | -42 | -70 | -24 | Tuber |
| 505 | 7.1519 | -44 | -48 | 60 | Inferior Parietal Lobule |
| 828 | 6.1881 | -14 | -28 | -20 | Culmen |
| 373 | 5.8574 | 54 | 20 | -10 | Inferior Frontal Gyrus |
| 61 | -5.6982 | 34 | 52 | -6 | Middle Frontal Gyrus |
| 3404 | -5.2579 | -4 | 40 | -6 | Anterior Cingulate |
| 416 | 4.5294 | -46 | 0 | -22 | Middle Temporal Gyrus |
| 83 | 4.0801 | 46 | -50 | 60 | Inferior Parietal Lobule |
| 69 | 4.0252 | -26 | -98 | 4 | Middle Occipital Gyrus |
| 140 | 3.8261 | 60 | 22 | 24 | Inferior Frontal Gyrus |
| 120 | 3.7406 | 68 | -40 | -6 | Middle Temporal Gyrus |
| 169 | -3.6648 | -10 | -82 | 30 | Cuneus |
| 55 | 3.5959 | 0 | -10 | -24 | No Gray Matter found |
| 476 | -3.5565 | 42 | -14 | 4 | Insula |

Table S1B. Top clusters of the T-maps using voxel-based features

| **Low intensity of sadness** | | | | | |
| --- | --- | --- | --- | --- | --- |
| **Volume** | **Max Int** | **X coor** | **Y coor** | **Z coor** | **Region** |
| 44605 | -7.5617 | 0 | 10 | 56 | Superior Frontal Gyrus |
| 39655 | 6.0716 | 46 | 46 | 24 | Middle Frontal Gyrus |
| 904 | -5.6803 | -6 | 46 | -8 | Medial Frontal Gyrus |
| 349 | 3.408 | -44 | 42 | 26 | Middle Frontal Gyrus |
| 216 | 3.2182 | -38 | 24 | 48 | Middle Frontal Gyrus |
| 501 | -2.5564 | 26 | -26 | -26 | Culmen |
| 354 | 2.1581 | 44 | 4 | -20 | Superior Temporal Gyrus |
| 143 | 1.8852 | -24 | 6 | -20 | Uncus |
| 184 | -1.8803 | -30 | 44 | 38 | Middle Frontal Gyrus |
| 60 | 1.8653 | -58 | 18 | 14 | Inferior Frontal Gyrus |
| 24 | -1.438 | -58 | -64 | 4 | Middle Temporal Gyrus |
| 17 | -1.3904 | -50 | -60 | 50 | Inferior Parietal Lobule |
| 14 | -1.287 | 50 | 32 | 16 | Middle Frontal Gyrus |
| 10 | 1.2665 | 58 | 16 | -6 | Superior Temporal Gyrus |
| 10 | 1.2547 | -60 | -12 | 42 | Precentral Gyrus |
|  | | | | | |
| **Medium intensity of sadness** | | | | | |
| **Volume** | **Max Int** | **X coor** | **Y coor** | **Z coor** | **Regions** |
| 58696 | 7.9986 | -22 | -4 | 34 | Cingulate Gyrus |
| 37426 | -8.3546 | -32 | -26 | 62 | Precentral Gyrus |
| 273 | -2.4724 | 36 | -42 | -24 | Culmen |
| 262 | -2.7742 | -6 | 46 | -8 | Medial Frontal Gyrus |
| 232 | -2.3295 | 26 | -94 | -6 | Lingual Gyrus |
| 187 | -2.2268 | 26 | -6 | -28 | Uncus |
| 169 | -1.9832 | 48 | -80 | 8 | Middle Occipital Gyrus |
| 106 | -1.4901 | -30 | 46 | 38 | Middle Frontal Gyrus |
| 102 | 2.0186 | -56 | -62 | -18 | Declive |
| 69 | -1.954 | 10 | -56 | -16 | Culmen |
| 55 | 2.045 | -16 | -92 | 0 | Lingual Gyrus |
| 52 | -2.1891 | 56 | -6 | -28 | Inferior Temporal Gyrus |
| 41 | -1.8766 | -6 | -58 | -14 | Declive |
| 39 | -1.5601 | 48 | -42 | 18 | Superior Temporal Gyrus |
|  | | | | | |
| **High intensity of sadness** | | | | | |
| **Volume** | **Max Int** | **X coor** | **Y coor** | **Z coor** | **Regions** |
| 99684 | 9.641 | 38 | -54 | -28 | Culmen |
| 3412 | 4.6867 | 46 | 48 | 20 | Middle Frontal Gyrus |
| 1803 | 4.0833 | -46 | 38 | 24 | Middle Frontal Gyrus |
| 43 | -2.6501 | 18 | -40 | -44 | Cerebellar Tonsil |
| 455 | 2.6454 | -48 | -50 | 52 | Inferior Parietal Lobule |
| 192 | 2.4105 | -16 | -16 | 20 | Caudate |
| 63 | 2.0323 | -30 | -74 | 38 | Precuneus |
| 1911 | 2.0011 | 2 | -30 | 10 | Thalamus |
| 13 | 1.979 | 16 | 38 | 48 | Superior Frontal Gyrus |
| 38 | 1.9177 | -64 | -20 | 32 | Postcentral Gyrus |
| 11 | -1.709 | 32 | -24 | -22 | Parahippocampal Gyrus |
| 29 | 1.6255 | -64 | -22 | 12 | Superior Temporal Gyrus |
| 11 | 1.514 | 22 | 66 | 14 | Superior Frontal Gyrus |
| 48 | 1.4665 | 26 | -28 | 18 | Insula |
| 12 | 1.4583 | -62 | -2 | -4 | Middle Temporal Gyrus |

In Tables S2A-B the regions were described according to the predefined anatomical template (Automated Anatomical Labeling, AAL template, Tzourio-Mazoyer et al. 2002).

**Table S2A. Top regions in the OC-SVM maps using regions based features**

| **Top regions for pattern based on the low intensity of sadness** | |
| --- | --- |
| **Weights** | **Regions** |
| 166.6191 | Temporal Polo Middle L |
| -150.1421 | Vermis 10 |
| -141.5444 | Cerebellum 9 R |
| -140.0101 | Cerebellum 9 L |
| -120.4585 | Cerebellum 10 R |
| 120.1527 | Temporal Polo Middle R |
| 112.6229 | Temporal Polo Superior R |
| -111.4114 | Cerebellum 10 L |
| 103.4911 | Temporal Polo Superior L |
| -100.8888 | Cerebellum 8 R |
| -98.7747 | Vermis 9 |
| 85.7475 | Frontal Inferior Orbital L |
| 82.4722 | Frontal Superior Medial L |
| 80.9826 | Cerebelum Crus1 L |
| -80.0876 | Cerebelum 8 L |
|  | |
| **Top regions for pattern based on the low intensity of sadness** | |
| **Weights** | **Regions** |
| 177.5059 | Temporal Polo Middle L |
| 151.8310 | Cerebelum Crus 1 L |
| 137.9672 | Cerebelum Crus 2 L |
| -136.6149 | Cerebelum 9 R |
| 132.4551 | Cerebelum Crus 1 R |
| 125.8022 | Temporal Polo Superior L |
| 124.5655 | Cerebelum 3 R |
| -120.3694 | Vermis 10 |
| 104.1197 | Temporal Polo Middle R |
| -103.7400 | Vermis 9 |
| 99.3243 | Cerebelum Crus 2 R |
| -93.3292 | Amygdala R |
| 84.6275 | Temporal Polo Superior R |
| -82.0555 | Cerebelum 9 L |
| -78.3169 | Cerebelum 8 R |
|  | |
| **Top regions for pattern based on the low intensity of sadness** | |
| **Weights** | **Regions** |
| 160.1872 | Cerebelum Crus 1 L |
| 156.5640 | Cerebelum Crus 1 R |
| 139.3083 | Cerebelum 6 L |
| 120.8605 | Cerebelum 3 R |
| 108.3616 | Temporal Polo Middle L |
| 105.5706 | Temporal Polo Superior L |
| -103.8854 | Cingulum Middle L |
| 102.4786 | Temporal Polo Middle R |
| -102.1654 | Paracentral Lobule L |
| 101.5555 | Cerebelum Crus2 L |
| 99.7323 | Temporal Polo Superior R |
| 93.1426 | Cerebelum Crus2 R |
| -92.9368 | Cingulum Middle R |
| -91.3523 | Frontal Middle Orbital R |
| -78.6727 | Cingulum Posterior L |

Table S2B. Top regions in the T-maps using regions based features

| **Top regions for pattern based on the low intensity of sadness** | |
| --- | --- |
| **T values** | **Regions** |
| -6.0869 | Motor Area Superior L |
| 4.6329 | Cerebelum Crus2 R |
| -4.3022 | Precentral R |
| 4.132 | Cerebelum Crus1 L |
| -3.9657 | Supperior Motor Area R |
| -3.6063 | Occipital Supperior R |
| 3.5473 | Cerebelum Crus2 R |
| -3.5105 | Postcentral R |
| -3.3394 | Parietal Supperior R |
| -3.3013 | Cingulum Middle L |
| -3.2383 | Cuneus R |
| 3.2055 | Temporal Inferior L |
| -3.1437 | Paracentral Lobule L |
| 3.0819 | Vermis 7 |
| 2.7191 | Frontal Middle L |
|  | |
| **Top regions for pattern based on the low intensity of sadness** | |
| **T values** | **Regions** |
| -5.3193 | Postcentral R |
| -5.2313 | Precentral R |
| -5.1824 | Supperior Motor Area L |
| -4.8896 | Supperior Motor Area R |
| 4.4232 | Cerebelum Crus2 L |
| 3.8520 | Angular R |
| -3.8414 | Parietal Supperior R |
| 3.7045 | Cerebelum Crus1 L |
| 3.5459 | Cerebelum Crus2 R |
| 3.3191 | Frontal Supperior L |
| 3.1537 | Angular L |
| 3.0906 | Temporal Inferior L |
| -2.9949 | Occipital Supperior R |
| 2.9915 | Vermis 7 |
| 2.8694 | Frontal Middle L |
|  | |
| **Top regions for pattern based on the low intensity of sadness** | |
| **T values** | **Regions** |
| 7.7011 | Cerebelum Crus1 L |
| 6.7796 | Occipital Inferior L |
| 6.5081 | Cerebelum Crus2 L |
| -6.1809 | Cingulum Middle L |
| 6.1318 | Cerebelum 6 L |
| -5.6692 | Cingulum Anterior L |
| 5.4983 | Cerebelum Crus1 R |
| -5.4897 | Cingulum Anterior R |
| -5.2033 | Cingulum Posterior L |
| -5.1992 | Cingulum Posterior R |
| -4.8821 | Paracentral Lobule L |
| -4.8667 | Paracentral Lobule R |
| -4.7409 | Cuneus R |
| -4.7359 | Cingulum Middle R |
| -4.7020 | Supperior Motor Area R |
